# Exhumations without (transitional) justice? Recovering the dead in Somaliland

**DOI:** 10.1016/j.fsisyn.2026.100666

**Published:** 2026-02-24

**Authors:** Lucia M.M.K. Elgerud

**Affiliations:** Uppsala University, Department of Archaeology, Ancient History and Conservation, Engelska parken, Thunbergsvägen 3H, Box 626, 751 26, Uppsala, Sweden

**Keywords:** Forensic anthropology, Mass grave exhumation, Somaliland, Ethnography, Transitional justice

## Abstract

Forensic investigations of political violence are pursued for retributive and restorative justice purposes, although the relationship between the two processes has been the subject of debate. Alongside these debates are conversations emphasizing the need for actor-, victim-, or survivor-centered agendas for transition after conflict. Using ethnographic data, this article accounts for the viewpoints of 74 Somalilanders who lost a family member during the political violence of the 1980s Siad Barre dictatorship. Discussions with families centered on recent mass grave exhumations, their knowledge of forensic anthropological investigations, and their wider views on local post-conflict processes. During interviews, families highlighted the need for exhumations, formal legal justice activities, and investments in physical and mental health institutions. While most family members want perpetrators tried through formal legal means, many would accept processes to inform the young, the international community, and the historical narrative about the atrocities, as accountability may be beyond reach. Moreover, some relatives are primarily focused on Somaliland's pursuit of international recognition, whereas others view investigations as volatile and believe they may spur renewed clan conflict. This article supports arguments brought forth in response to post-conflict investigations for over 20 years: family needs are complex and must be pursued during forensic work using survivor-centered approaches. Furthermore, this article shows the complex and context-specific needs of families of the dead in Somaliland. Arguably, these needs should be central to further post-conflict work in the region, and they can also inform emerging transitional justice efforts across North Africa and the Middle East.

## Highlights

• Families of the dead in Somaliland have needs that are not being met by recent mass grave exhumations.
• Families of the dead are generally supportive of mass grave exhumations but a majority are also looking for formal legal justice processes to hold the perpetrators accountable, preferably in international courts although following Shari'a principles.
• Since few family members of the dead believe their loved ones' killer can be held accountable (since the conflict took place over 30 years ago and Somaliland is not internationally recognized) many would instead accept documentation of atrocities to inform the young, the world, and the historical narrative.
• There are concerns amongst Somaliland communities that forensic investigations through mass grave exhumations will lead to renewed conflicts between clans.
• The Somaliland case shows the continued need for locally specific survivor-centered approaches to understanding post-conflict needs in order to support transitional justice processes that respond to local experiences, socio-political contexts, and ethnic divisions.

## 1 Introduction

Somaliland, a self-declared autonomous region in the Horn of Africa, began conducting human remains recovery operations in the early 2010s. Supported by forensic anthropologists from Peru and students and practitioners from other parts of the world, several mass graves have been exhumed to analyze, identify,^1^ and rebury the victims. The graves are associated with dictator Mohamed Siad Barre, who ruled Somalia with an iron fist from 1969 until he was overthrown in 1991. The mass grave exhumations and the subsequent analysis of human remains have been led by a Somaliland governmental organization, which, while initiated by concerned relatives, has been pursued with little family input or community sensitization, despite an increasing emphasis over the past couple of decades on the need for family involvement in the forensic process [[1], [2], [3]]. The study presented here asks how family members find the completed human remains recovery operations, including their knowledge level of the forensic investigation and their viewpoints on the dead, the graves, and what to target in ongoing post-conflict work. Somaliland remains a relatively underdeveloped country, with a large proportion of the population illiterate and socioeconomically disadvantaged. Moreover, the effects of the war are palpable throughout the country, including in Hargeisa, the capital of Somaliland. The city is rife with buildings displaying bullet holes and ruins following mass bombings by the government via contracted fighter-jet mercenaries [4,5].

The role of forensic investigations in the form of human remains recovery after conflict and as part of transitional justice frameworks has been under scrutiny for some time. While forensic processes, not least in the form of mass grave exhumations, have indubitably supported legal mechanisms as evidence collected has been called upon to hold perpetrators accountable [6,7] debates also focus on forensic investigations’ role outside of formal legal processes such as emphasized within the International Committee of the Red Cross (ICRC) Humanitarian Forensic Action (HFA) approach. Such frameworks have been criticized for failing to meet family, survivor, and victim needs that center on due process [1,8] although proponents emphasize the potential to restore dignity for the dead, fight ambiguous loss, support families right to truth and to know what happened to the dead, and for the opportunity to rebury the dead in culturally proper ways [[9], [10], [11]]. Forensic investigations that are conducted outside of official political and legal negotiations have also been argued to support grassroots movements toward reconciliation and pseudo-legal processes as communities have come together to process past violence when human remains have been unearthed and violence documented and recounted [[12], [13], [14]].

Over 20 years ago, Stover and Shigekane urged forensic scientists to advocate for the dead as much as for the living, as they proclaimed how families need to “be actively involved in the legal and humanitarian efforts to locate, exhume, rebury, and memorialize the dead” [1]. Moreover, continued emphasis is placed on actor-, victim-, or survivor-oriented approaches to forensic investigations during and after conflicts and political violence, as well as to wider transitional and transformative justice frameworks [2,3,[15], [16], [17], [18], [19]]. With the emphasis on family needs well established and the necessity of understanding the complexities underlying political, cultural, legal, and inter- and intra-group tensions, the remaining task is to gauge context-specific needs related to forensic investigations. This work attempts to do just that in Somaliland, taking into account the unique geopolitical context; the complex clan component that undergirded the conflict and continues to affect Somaliland society; and the state of exhumations, given the limited resources and the conflict's antiquity. The exhumations were also assessed with respect to their spiritual and religious underpinnings, as related to the handling and reburying of human remains in Muslim communities in general and in Somaliland in particular, although this, along with the more social-theoretical and necropolitical/-governmental components of the work, falls beyond the scope of this paper.^2^

Ostensibly, the work in Somaliland has not been conducted alongside the wider community of families of the dead, nor have there been wider sensitizations to inform communities of the forensic process and the scope, methods, and goals of the exhumations that were ongoing between 2012 and 2018 [4,20,21]. Reasons for these shortcomings will not be further analyzed here, but they can largely be attributed to a lack of institutional motivation, resources, and organization, and to their presumed relation to Somaliland's fragile peace (see footnote 2 for further reading on the topic). Due to the seeming lack of wider family involvement in the human remains recovery operations in Somaliland, a study was conducted to understand the viewpoints and sentiments of families of the dead as related to the forensic investigation and wider post-conflict needs. The work focused on family understanding of the process, which turned out to be very slight, and their needs associated with the dead, the graves, and the Siad Barre era violence. Discussions with family members addressed a range of processes that can form part of post-conflict efforts, including legal processes, reparations, memorialization, and truth-telling, as probed by the interviewer and raised by the relatives themselves.

As will be explored here, family members primarily emphasized the need for formal legal processes and accountability, preferably grounded in Shari'a principles. Although with little faith that the perpetrators could be found or held accountable, families also emphasized the need for documentation to remember what happened, truth-telling, informing the historical record, social support for the survivors, individual identification of the dead, and, to some, international recognition for Somaliland. The forensic investigation also sparks fears related to re-traumatization and renewed conflict, which families relate to both truth-telling, reparations, and the exhumations themselves. These responses contribute to a temporally and thematically deep body of knowledge, emphasizing the complex nature of forensic investigation, which cuts across and reconfigures what may be classically defined as retributive or restorative justice mechanisms. Each context in which forensic investigation has been, or is intended to be, incorporated requires an understanding of family needs and sentiments, informed by geopolitical, cultural, and conflict-related specifics, and preferably an integrated approach to forensic investigation [2,3,12,19,22,23]. This article provides a snapshot of needs specific to Somaliland, which can serve to support families of the dead in three ways: 1. By pinpointing locally specific policy requirements and areas to be further investigated during any continued forensic investigation in Somaliland, 2. By providing additional evidence of overlapping restorative and retributive justice needs after conflict as related to forensic investigation, yet again highlighting the necessity of context-specific gauging of family needs and community sensitization, and 3. By pinpointing areas of concern for families that may be relevant to comparable post-conflict contexts, specifically in countries in North and East Africa and the Middle East, as they seek to incorporate transitional justice mechanisms.

## 2 Theoretical framework

During transitional justice efforts, communities may seek redress for atrocities committed under an oppressive government as a country shifts from authoritarian rule to a democratic society that respects individual freedom and human rights [24,25]. Forensic anthropologists have been integral to the transitional justice process by assisting in finding individuals who have been detained, disappeared, and executed, and documenting criminal behavior through the exhumation of burials and the analysis of human remains [21,23,[26], [27], [28]]. Recovering human remains has been assumed to contribute to reconciliation and healing alongside legal or institutional transitional justice goals. Finding the missing, caring for the dead, and investigating their deaths can be important processes for the families of the missing and killed, and burying loved ones can allow for fundamental spiritual and religious mortuary practices [1,11,26,[29], [30], [31]].

Human remains can underscore several processes after times of violence, as there are many family needs that may be related to the dead, both on a surface level and in more complex ways. The families of the dead may be searching for answers to whether their loved ones are dead or alive, to find out what happened to them through forensic analyses, and to rebury them according to local burial practices. Furthermore, families may seek to hold perpetrators accountable and to have human remains analyzed to support evidence collection for formal legal processes [1,11,26,[31], [32], [33]]. The dead can also impinge on the living in more complex ways, i.e., as in northern Uganda, where the dead are causing spiritual disturbances, or as in Nepal, where women who do not hold a death certificate are socially and financially tethered to their disappeared husbands' families [2,34,35]. However, families may have opposing views on how human remains should be dealt with, including whether remains should be exhumed at all, and families may be divided on ideological or political grounds. Following the Argentinian conflicts of the 1970 and 80s, some family groups were eager to find the disappeared, dead or alive. Other family groups actively opposed locating and identifying the dead as it would not bring their loved ones back, while it would signify an end to the state's responsibility [30,36]. Hence, understanding family needs is not simply a matter of taking stock or securing the support from some families over others to recover the dead. Needs can intersect with legal, cultural, and opposing views of family and ethnic groups, including views that may themselves be volatile during post-conflict rebuilding and reconfiguration of society. Human remains can serve as powerful symbols after conflict, invoked to support political narratives concerning culpability and the appropriate post-conflict order by those in power [30,37,38].

An exclusionary focus on humanitarian support or the dignity of the dead can serve to lose track of family needs that may themselves be related to political order, power, and more complex living-dead interactions. The Forensic Unit of the International Committee of the Red Cross advocates for a Humanitarian Forensic Action (HFA) to aid families through forensic means, though on neutral grounds. According to Cordner and Tidball-Binz: ‘Humanitarian action itself is defined by the ICRC as a range of activities that seek to alleviate human suffering and protect the dignity of all victims of armed conflict and catastrophes, carried out in a neutral, impartial and independent manner, free of charge and framed under International Humanitarian Law ‘[10]. This framework has been criticized for failing to account for the many ways in which forensic science intersects with broader legal and political contexts. According to the ICRC, HFA is said to derive from the work of the Argentine Forensic Team or EAAF. However, during EAAF's early work with families of the dead in Argentina, they continuously recorded evidence that could be used for legal purposes once amnesty laws were overturned [8]. While the work of the ICRC has undoubtedly supported families' search for the missing, ignoring the interrelatedness of family needs and judicial process or political underpinnings can have detrimental outcomes. In contrast, Kovras identifies what he calls the Forensic Cascade – the implementation of forensic science in transitional justice contexts – as supporting restorative as well as retributive justice processes in different settings [6]. Forensic investigations have lent support to humanitarian forensic work, truth-seeking, and the documentation of atrocities, as well as to retributive justice through evidence collection, he argues. In this way, the forensic cascade has been imperative to transitional justice frameworks as it has helped to delink the ‘trade-off between justice initiatives and efforts to consolidate peace in fragile transitions’ [6]. However, as will be seen here, what is at stake in Somaliland is perhaps rather the forensic investigations' simultaneous impact on families urging legal processes (with clear agendas for their implementation as well as their substitution) and on the tenuous peace since the fall of the Siad Barre dictatorship.

For over 20 years, scholars have argued for further community integration before, during, and after forensic investigation, and for consideration of how forensic processes intersect with cosmological, political, legal, and social concerns [22,23,39]. Despite this emphasis, there remains a lack of family and survivor involvement and of community sensitization that adequately serve families while managing expectations. Transitional justice frameworks have historically been driven by those in power for the purpose of supporting state building; however, a grass-roots perspective is needed to understand victims and survivors' needs, perhaps even root causes of conflict, and whether forensic processes or other transitional justice mechanisms can support those needs [[1], [2], [3],^19^,^40^,^41^]. This work necessitates careful deliberation with families and gauging of experiences, sentiments, and appropriate cultural practices throughout the forensic process [2,3,19,42,43]. An effort of this nature was conducted by Simon Robins in his research with families of the disappeared in Nepal. Here, he employed a victim-centered approach to investigating the concerns of families of the missing in post-conflict Nepal to inform the transitional justice policy that had primarily focused on ‘justice’ as advised by local elites. The study showed that the government adopted a top-down approach, focusing primarily on civil and political human rights in the aftermath of the conflict, rather than on social, cultural, and economic rights, which families prioritized [2].

In light of these developments, this article looks at the context of Somaliland, where forensic science has been employed by a government institution to exhume and rebury the dead, although without wider family input or community sensitization. Hence, this study asks whether the model pursued in Somaliland is supported by families of the dead and what family needs look like as related to the dead and the graves. To answer these questions, a qualitative study was conducted amongst family members to understand their needs and their experiences with forensic science, using a survivor-centered approach. The terms victim and survivor are often used interchangeably in transitional justice literature, although the victim-perpetrator binary assumes a neat distinction between those who experience and those who perpetrate harm, see for example [18,44]. In this article, the term ‘survivor-centered’ or ‘family-centered’ is used to refer to the family members of those who were killed by Former Somali President Siad Barre to avoid qualifying the victim-perpetrator binary. Building on the framework used by Robins to understand families' needs in post-Maoist insurgency Nepal, this qualitative research accounts for the viewpoints and needs of 74 family members of those who died in present-day Somaliland under dictator Siad Barre's regime [2].

## 3 Background

### 3.1 History of the Somaliland conflict

Army general Mohammed Siad Barre seized power over Somalia through a bloodless coup in 1969. Siad Barre and his new government, the Supreme Revolutionary Council (SRC), swiftly altered the political system and introduced ‘scientific socialism’: a Marxist-Leninist economic model coupled with an emphasis on Islamic values. SRC leaders were appointed to positions of power in the administration, while former officials were imprisoned and opposing political parties were banned [5,[45], [46], [47]]. Siad Barre soon established the National Security Service (NSS), the military police, and the National Security Court - institutions that were granted unlimited power to arrest, prosecute, imprison, torture, and execute any dissidents [5,[46], [47], [48]].

The north, an area populated primarily by the Isaaq clan family, was particularly targeted during this time. The Siad Barre regime implemented increasingly authoritarian laws in the north, including provisions prohibiting more than two people from conversing in public [49]. The inhabitants were made aware that they were watched at all times and that they could not disapprove of the government: ‘Rather than providing protection for its citizens, the state became a tool of repression ‘as defined by Bradbury [5]. In 1981, diaspora communities from London and Saudi Arabia, seeking to rebel against the Somali government, came together to form the Somalia National Movement (SNM) [5,50,51]. Support for the SNM grew rapidly in northern Somalia, and the government responded by increasing the police force and NSS presence. Any civilian could be arbitrarily accused of being an SNM member or supporter and be punished accordingly [5,46,52].

After years of clashes with the army, the SNM attacked government troops in Hargeisa, the northern capital, and the town of Burco in May 1988, to which the government responded by sending planes from Hargeisa airport to aerially bombard the city. Simultaneously, the government soldiers went through Isaaq houses, killing thousands, allegedly in the search for the SNM. Areas that were thought to harbor human life were bombed, killing tens of thousands and forcing around half a million inhabitants to flee the city [5,46,50,53]. Civil war ensued until early 1991, while revolutionary dissidence escalated in southern Somalia. Siad Barre eventually fled Mogadishu in January 1991. As southern rebel movements claimed power in the south, the SNM declared Somaliland an independent territory, and soon the displaced population returned to Hargeisa and the rest of the region [47,50,52]. It is not known how many people were killed in the 1980s by the Siad Barre regime in what is presently Somaliland; estimates have ranged between 5000 and 50,000, although some local officials believe that the number is closer to 100,000 or even 200,000 people [4].

### 3.2 Somaliland and transitional justice

The self-proclaimed autonomy of Somaliland was established in May 1991 at a conference in Burco. The conference aimed to build peace between the Isaaq and various local non-Isaaq clans that supported the government forces during the war. Several conflicts occurred after 1991, and additional peacebuilding conferences were held in 1993, 1994, and between October 1996 and February 1997 [47,54]. During these conferences, it was decided that Somalilanders who committed crimes during the war would be given a general amnesty for the sake of peace and unity. Since 1997, the region has experienced near-continuous stability (though with some tension in the borderlands with Puntland), attributed to the conferences and localized approaches to peacebuilding [50,54,55].

In May 1997, flooding from heavy rains scattered the sand atop buried human remains in Hargeisa, exposing several mass grave sites associated with the Siad Barre regime atrocities. Later that year, Physicians for Human Rights (PHR) was appointed by the United Nations High Commissioner for Human Rights (UNHCHR) to conduct preliminary investigations. PHR submitted a report to the UNHCHR in 1998 in which they recommended further investigations into the graves and for all mass graves in the area (a minimum of 92 according to PHR's investigation) to be surveyed for future exhumation [56]. Soon after the PHR investigations, the government of Somaliland established the War Crimes Investigation Commission (WCIC) to investigate the Siad Barre era atrocities. The WCIC has since collaborated with the San Francisco-based Center for Justice and Accountability (CJA), who have filed lawsuits in the USA on behalf of Somali victims. The CJA has pursued litigation under the tort statute against several offenders from the Siad Barre regime in the U.S. federal court system. The defendants have included the former Prime Minister of Somalia, General Mohammed Ali Samatar, the former Head of the Fifth Brigade of the Somali Army, Colonel Yusuf ‘Tukeh’ Abdi Ali, and the former Head of President Siad Barre's Secret Service, Colonel Abdi Aden Magan.^3^

In 2010, the WCIC and the CJA, supported by diasporic Somalilanders living in California, invited the Equipo Peruano de Antropología Forense (EPAF) to conduct mass grave exhumations in and around Hargeisa. EPAF has since engaged in human remains recovery operations across Somaliland, manned by international students through semi-annual field schools.^4^ The operations have been met with mixed sentiments and occasional hostility, largely spurred by the lack of community sensitization on the work [4]. The exhumations have been conducted without broader family participation, and there is little information available to local communities about the scope and objectives of the investigations. To understand the impacts of the exhumations, including whether the recovery operations meet family views and needs, a qualitative study was conducted in 2019 amongst 74 family members of the dead [20].

## 4 Study methodology

This study was conducted as part of a wider, qualitative research project aimed at understanding the impacts of human remains recovery operations in Somaliland from the perspective of family members of those who were killed by the Siad Barre regime. The research consisted primarily of interviews conducted over five months in 2019. Local collaborators acted as discussants and consultants throughout the research and assisted as translators for semi-structured interviews. All interviews used a predesigned, but flexible interview protocol and lasted around 60 min each. The flexible protocol meant that, although most interviews probed similar themes, some topics were discussed in greater depth depending on the interviewee's interests and viewpoints. Primarily, those living in larger cities are represented in the data, as interviews were conducted in Hargeisa, Berbera, and Boroma. Interview locations were based on funding, time, and travel restrictions during the research.

Family members that were interviewed ranged from socioeconomically affluent businessmen, lawyers, and ministers to the less affluent (e.g., those working at local markets) and those unable to work due to disabilities and material losses, typically war-related issues. The majority, however, were poor, and many were illiterate. All but eight of the 74 were of the Isaaq clan, and the others were from clans that are in the minority in Somaliland, including Samaroon, Hawiye, and Gaboye. Thirty-four of the participants were female, 40 were male, and all were adults. The interviews were aimed at understanding family members’ views on the mass grave exhumations that have been ongoing under the WCIC, and family needs related to the human remains. Families emphasized several processes that they found integral to the recovery operations, but that go beyond the exhumation itself, such as the need for former legal processes and remembrance through documentation. In response, interview questions focused on further post-conflict processes and transitional justice mechanisms, such as the need for evidence collection for fighting impunity, appropriate courts or punitive measures for perpetrators, the need for reparations, and whether they were interested in further truth-telling, which may or may not be supported by forensic investigation [6]. The interviews were audio recorded, transcribed, and coded using NVivo software to analyze central themes, frequency of specific responses, and for identifying relevant quotes.

All survivor-centered views on how to best deal with past violence constitute a ‘snapshot’ of needs, which may have changed since data were collected for this study [2]. Moreover, since the Somaliland atrocities took place over 30-years ago, those needs are also affected by fading memories of atrocities and trauma. However, while some have chosen to leave the atrocities in the past [57], many have vivid memories and open emotional wounds from the war – traumas that many are seeking to work through and heal. Since little has been done in Somaliland to deal with the violence of the Siad Barre era and its perpetrators, families were generally encouraging of the research and further work related to the conflict and the graves. Several interlocutors stated that the study should expand to reach all who lost someone during the Siad Barre era violence, since few have been able to share their stories or seek justice for the crimes committed against their families.

## 5 Results

The following sections outline the needs of Somaliland families, beginning with their views on exhumations and identification efforts, and whether they would support DNA methods and capacity building in Somaliland for this purpose, followed by family members' views on collecting evidence from the remains. Subsequent sections focus on needs highlighted by families centered on formal legal justice efforts. Despite contemporary emphases on recovering human remains for purely humanitarian (and non-judicial) reasons [10], families were primarily focused on holding perpetrators accountable for the deaths of their loved ones. Moreover, families had clear agendas regarding who should be held accountable and how they should be held accountable, typically centered on Shari'a-based legal processes. These results are presented in the following sections, followed by economic needs and viewpoints among family members, the topic of remembering and publicly discussing the violence of the Siad Barre era, and the potential utility and drawbacks of truth-telling forums.

### 5.1 Need for human remains

Burial practices in Somaliland follow common Islamic burial provisions, including washing, shrouding, and burying the dead facing Mecca during prayers. The dead should all be buried in a similar, austere manner, without lavish graves or tombstones. As many of the war dead were buried clandestinely or died while the city was being bombed, they were not given traditional mortuary care following local customs. By identifying the dead and reconnecting them with their families, survivors may be able to care for their dead and bury them properly – or so the theory goes. However, communities may find that the recovery of human remains is not necessary on spiritual, political, or psychosocial grounds. In some contexts, rituals have been conducted to target loss and mourning even in the absence of human remains [58,59]. Hence, family members in Somaliland were engaged in discussion regarding whether they saw a need to exhume and rebury the dead. These discussions intersected significantly with religious viewpoints regarding burial and locally observed Islamic restrictions on opening a grave without a clear necessity, details of which are beyond the scope of this paper. See Elgerud [20] for more details. To date, no large-scale molecular methods have been incorporated to identify the dead in Somaliland, primarily due to funding restrictions. For this reason, discussions with family members who highlighted the need to find the dead also included discussions regarding individual identification of the dead and their views on and knowledge of DNA methods.

A total of seven individuals out of 74 (∼9 percent) were unsupportive of forensic investigation, and two were unsure. Of those who opposed the investigation, two were Isaaq, and the other five were from the Somaliland minority clans, Gaboye and Samaroon. However, the other non-Isaaq individuals were supportive of some aspects of the forensic work, albeit with some hesitation. Similar hesitancies were also observed among many Isaaq family members. For example, some believed that investigations should not be conducted unless remains had already been unearthed by floods, as in the 1990s, but once remains were exposed, they should be investigated, identified, and properly reburied. Others believed that neither identification nor analysis was important, but that forensic investigation should still proceed for political reasons. Of 74 family members, 68 directly engaged with the question of identification, and 60 considered identification important. Many of these family members believed individual identifications should be prioritized during the forensic work, if not prioritized over other social needs in general, even beyond the mass grave investigations. The majority of the 60 individuals (∼88 percent) found identification to be an important, if not the most important, facet of human remains recovery operations and would support DNA methods for this purpose. The eight (∼12 percent) who did not stress the need for identifications instead stressed the redundancy of spending time and money on finding those who died over 30 years ago, those who have already been assumed to be dead, and their remains lost. Despite their disinterest in identifications, the eight still stressed the need to shroud the dead and bury them in individual graves, although some also believed it would create hostility if time and funding were invested in identifications.

### 5.2 Human remains & evidence

Since few family members knew any details about forensic analyses and what human remains could reveal (or not), time was spent on going over the basics of forensic anthropological analyses during the interviews (see Elgerud [20]). After these conversations, many expressed how they found it important to know all details about a loved one's death and their remains, although others believed it would be too traumatizing to learn this information or found it redundant since it would not bring the dead back. Some individuals felt that analyses were important for evidentiary purposes, though it was not important to share this information with family members, as it could be distressing. For example, family members who had witnessed the deaths of their loved ones were typically uninterested in scientifically corroborating their stories – they already knew what happened – though many of them believed evidence collection for the purpose of legal proceedings would still be useful.

Some family members were unfamiliar with the concept of evidence collection, whereas others were more familiar with it. Some were quick to point out that they did not find human remains recovery and reburials important unless there were also analyses to look at what happened to the dead, whether it was for evidence collection for legal purposes or simply to know what happened to a loved one. For example, according to one man, trials were necessary to hold perpetrators accountable, and evidence should be collected to ensure they were as fair as possible. The man was eight years old when government soldiers arrived at his father's land, dragged his father to a nearby field, and shot him dead in front of his young son:

‘Although I don’t have the power to judge them, the only thing that would give justice is through international courts. If the courts receive all the information on what happened here, I am confident that the courts would judge these people properly - that’s the only thing that would make me happy.‘^5^

When asked if he thought that evidence from the graves should be used in the courtrooms, if possible, the man stated that:

‘It could maybe help, it may not help, but it is better than to not give any information because although what I am saying is real, I do not want to accuse anyone who is innocent - everything that I have told you is 100 percent real but sometimes the truth is hidden, but I believe one day this information will help us.‘^6^

### 5.3 Justice

A majority (85 percent) of family members focused on issues of justice and the need to hold perpetrators accountable for the deaths of their loved ones. When asked what justice for the Siad Barre regime killings would mean to them, a majority discussed formal legal justice. Responses ranged from punitive measures to exposing what happened and ensuring that the public knew the perpetrators were criminals. Some family members assumed that the primary purpose of the recovery operations was ‘justice’ for the crimes committed, specifically punitive measures and the holding of perpetrators accountable. For example, one 60-year old woman had heard that “those human rights activists are to exhume the graves and the victims will get their justice as the perpetrators will be put on trial.‘^7^ Although not all family members were under the impression that forensic investigation would lead to legal proceedings or to some form of justice, many wished it would.

There were some views on justice that were further removed from legal processes and evidence collection. For example, some family members equated justice with formal separation from Somalia through international recognition; with the claim that the current peace in Somaliland was all the justice they needed; or with the demand for financial reparations to address the loss of breadwinners, livelihoods, or children's education. One man's perspective was one of the more extreme: ‘I believe that the only justice is to be separated from those who did us to this, this kind of genocide to us, so we have to be separated, two separate countries. That would be justice to all Somaliland.‘^8^ He believed that recognition was more important than any legal proceeding or other transitional justice measure. In contrast, other family members were adamant that, while recognition was a significant goal, most importantly, the perpetrators needed to be put on trial for their actions.

Of 74 family members, 63 (85 percent) emphasized the need for legal processes to target perpetrators, whereas the remaining 11 were ambivalent or not interested in formal trials. Eight of the 11 did not believe that there should be a legal process, mainly because it could lead to tensions between the Isaaq and the clans who supported the government under Siad Barre. Four non-Isaaq clan members were of this opinion. Similarly, some non-Isaaq clan members mentioned that they had an interest in seeing their loved one's killer held accountable, but that they would rather leave it in the past since trials would lead to more conflicts, they believed. The perpetrators in these contexts were typically SNM fighters, who have been hailed as heroes by the Isaaq majority. However, the SNM fighters have also been accused of extrajudicial executions. There are even reports of SNM attacks on United Nations Development Program (UNDP) refugee camps. Fighters reportedly executed government officials and pro-government civilians that they captured during the war, leading many non-Isaaq clan members to want to seek justice against SNM fighters [5,46,60]. Given the large Isaaq majority and the national veneration of SNM as heroes, these viewpoints could be seen as inflammatory, prompting hesitation toward the forensic investigations, as some of the interviewed individuals believed they would undoubtedly lead to more clan conflict. In addition, several Isaaq and non-Isaaq clan members believed that there was no point in pursuing the criminals since the war occurred so long ago and many of the perpetrators have since died. Others believed that the responsible colonels and officers were ‘living their lives’ in Somalia, Europe, or America, and that this was unfair since it should be known that they committed horrible crimes, something that could perhaps be achieved through forensic work. As such, these family members were confident that some form of justice could still be achieved through forensic investigations and the announcement of the perpetrators' culpability, with or without formal legal processes.

### 5.4 Accountability

A total of 40 out of the 74 family members (∼54 percent) discussed accountability and who should be held accountable for the deaths of their loved ones. These discussions tended to focus on whether the person firing the gun, or other weapon used for execution, was the ultimate perpetrator, or whether they were simply foot soldiers who had to carry out orders given to them by higher members of the army, such as officers and commanders. Out of the 40, 26 believed that only those giving orders to execute were to be held accountable. These 26 family members generally reasoned that the soldiers were made to carry out orders and had no choice, and were thus not accountable for the deaths.

Out of the 40 individuals who focused on accountability, 14 believed that all who took part in executions during the war should be held accountable for their actions. Additionally, one man explained that the airmen who bombed Hargeisa in 1988 needed to be held accountable for him and his family to ‘get their justice’, since the pilots were responsible for his family's deaths. Some specified that those who only carried out orders should be given a lesser punishment, though all were culpable if they took part in the killings and should be judged according to the law. Non-Isaaq clan members were typically less interested in legal proceedings; hence, the discussions did not address their preferred legal forum or their views on forensic investigation for evidence collection. Out of the non-Isaaq clan members who were interested in trials, none believed any trial in Somaliland would treat their cases fairly since the crimes were perpetrated by members of the SNM. Some argued that Somalilanders could not be held accountable because of the amnesty granted at the 1990s reconciliation conferences, so there was no point in pursuing further legal action despite their lingering feelings of injustice. According to one woman, a medical professional of the Samaroon clan, there should not be any investigations into the dead or crimes committed during the war. Although her uncle was killed and her family had to flee their home and land, she believed that:

‘ … what has already been done is in the past, it has gone away, it is not important to look into now. There is no importance to talk about what has happened before. Trouble will be created if people start investigating what happened during that time.‘^9^

### 5.5 Preferred legal forum

The question of where and how the perpetrators should be tried, if at all, was discussed with 58 family members; three would prefer a domestic court and were not interested in international criminal proceedings. An additional four individuals would simply like to see the perpetrators tried, regardless of the court, and the remaining 51 individuals were either supportive of international trials, or of a combination of legal forums that included international proceedings. About half of the 51 individuals who preferred international trials would support domestic proceedings but did not believe this would be possible for some time, or until Somaliland had gained international recognition.

Most family members were familiar with the legal cases against Ali Samatar and Tukeh in the USA and mentioned these trials while discussing accountability. Families were generally favorable toward these trials and would support additional prosecutions against the Siad Barre commanders in the USA or elsewhere. A few family members pointed out that it was too far to travel to America if they wanted to participate, so proceedings should take place nearer by – perhaps Ethiopia or Kenya, they proposed. Family members were more favorable toward international rather than domestic trials for two main reasons: 1. They did not believe that the Somaliland legal structure was well enough established to deal with such high-profile cases, and 2. Since Somaliland is internationally unrecognized, other countries would not be able to extradite and relocate any perpetrator to the territory for trial. Few were opposed to domestic trials alongside international processes, with many indicating that they would support any court capable of targeting the criminals. Several family members also mentioned that once Somaliland is recognized, domestic courts could do more to address grievances arising from the war, although this could take a long time, and perpetrators should be dealt with as soon as possible; thus, international trials were favored.

### 5.6 Punitive measures

Suitable punitive measures for perpetrators were discussed with 45 family members, 14 of whom believed that the death penalty was the only suitable punishment for those who ordered executions. An additional 16 family members believed that the perpetrators should have treated them as they treated others. Ten individuals did not specify a sentence; instead, they believed that the perpetrators should be judged according to the law and that they would personally accept whatever punitive measure the courts imposed. Two additional individuals believed that the most important punishment was to ensure that perpetrators recognized that they were criminals. One individual believed the perpetrators should be imprisoned, and two individuals believed it was up to the families to decide on the sentences, in accordance with Shari'a customs. Several family members also explained that they would be happy as long as the perpetrators received a very severe punishment.

Conversely, some family members did not believe that the perpetrators deserved to die for their actions. To one woman who was a baby when her father was killed by the regime, the perpetrators should be imprisoned:

‘I think the perpetrators should pay compensation and go to jail. I don’t want them to die - me personally, I don’t want them dead, but they have to face the justice and to let them know that what they did was awful! … those people have to know that they are guilty, that they did something bad to the people. So, instead of enjoying their families and you know, like, having a good life. They need to be brought before a court, and see what they did, and feel it.‘^10^

### 5.7 Shari'a

Survivors often discussed religious ways of dealing with the injustices and the trauma of the war, and family members would typically state that the Somaliland Islamic criminal law-based courts would offer the most appropriate punitive measures for the Siad Barre regime offenders, even if they did not believe the courts were currently strong enough to handle these cases. Somaliland has three interrelated legal systems: Somali customary law, state (formal) law, and Islamic law [55]. The three systems are interrelated, and families can seek justice and counsel from either of the three institutions. However, there are several complexities and inherent flaws in this system, partly because few legal scholars are trained in all three codes, as described during conversations with legal scholars in Somaliland. In addition, colonization and the incorporation of state law in many Islamic communities have adversely altered the fluid and contextual nature of Shari'a [61,62]. According to Hussin, Islamic law can refer to the more black-and-white Shari'a as codified in legislation, but it can also refer to the more culturally applicable Islamic law of society as passed down within the family and community [61].

While families in Somaliland typically indicated that Shari'a-based law, as observed in local Islamic courts, should be incorporated while holding perpetrators accountable, no further discussion was held regarding their views on Shari'a as a customary or legal framework. In sum, families were primarily interested in international legal action and in punitive measures grounded in Islamic criminal law as recognized in Somaliland. However, the possibility of applying Somaliland Shari'a-based law in an international trial or criminal tribunal is beyond the scope of this research.

### 5.8 Economic needs

Many families in Somaliland lamented their loss of incomes and livelihoods as a result of the killings under Siad Barre and the bombing and subsequent exodus from Hargeisa. Grandmothers had been forced to care for grandchildren from one or more of their children, despite their old age. Women who had themselves been physically impaired by the war were forced to make a living to support their children, as their husbands had been killed and they had been separated from their parents and other support networks. Despite these circumstances, few family members stressed the need for monetary reparations, and the few who did were confident that no compensation could be obtained and that no defendant or institution would pay it. The need for compensation was discussed with 36 family members; 15 were supportive of reparations to some degree, and 18 were not. Three individuals stated that they would support compensation if appointed by the courts. Many of those who were not supportive of compensation stated that they did not believe receiving reparations was a possibility, partly because there were ‘too many victims’. Some believed that compensation was not important in light of losing a loved one. Many supporters of compensation also stated that they did not believe they would ever receive such funds. The Siad Barre government was long gone, and many of the perpetrators had died, so they did not believe that there was any point in hoping for compensation. A few individuals mentioned that compensation would be desirable, but, more importantly, they would like Somaliland to be formally separated from Somalia and internationally recognized.

Only about a third (5 people) of those who supported compensation considered it the most important facet of justice for the crimes committed; some also believed that, alongside compensation, the perpetrators should be imprisoned or executed. The remaining two-thirds were hesitant when discussing compensation, despite being in favor. These family members instead framed compensation as ‘financial help’, or support for education and other livelihood-sustaining assistance to make up for time and opportunities lost to the war. This was expressed by one man whose father was killed when he was a child:

‘Our families, like my mother, they suffer, she has had a hard time supporting my life, my education, how to get my basic life. There is not any support from the government, or from anyone else, but my mother deserves support to be able to look after her family.‘^11^

Similarly, during research in Nepal, Robins [2] found that families emphasized the need for economic support to compensate for income lost due to the disappearances. However, several family members were uncomfortable with assigning a monetary value to their loved ones' lives and instead referred to the economic support as ‘relief. ‘[2].

All but two of the 36 individuals who discussed compensation were Isaaq. Of the two non-Isaaq clansmen, one supported compensation for their losses, and the other did not. The latter was a man of the Samaroon clan. He believed that his generation had been deprived of education and opportunities to succeed and support themselves. The man believed that forensic investigation could be useful in many respects, and he was interested in seeing his family members exhumed and reburied if possible. However, he was not interested in any legal process since he found it more important to focus on those who are alive, moreover he believed that compensation could bring more conflict:

‘If you talk about compensation, there could be more problems. Maybe if compensation is given by one side, a different group will also ask for compensation. It will be too much! Nobody can cover it, so it’s better to forget, as it is now. There are a lot of things that are more important. What happened was a big, big conflict, which causes a lot of death. If we now talk about investigations and compensation, it will create another trauma, which will be bigger than before, especially if we focus on compensation. Everyone will want money; if you give to one part, another part will fight. Even more people will die from this than died in the past.‘^12^

### 5.9 Remembering

To survivor families in Somaliland, justice was typically raised as a chief concern when discussing the remains of their loved ones. In addition, the topic of justice was repeatedly discussed within the context of memory and history. Whether due to the time period of the conflict (the war taking place over 30 years ago), the difficulty of holding perpetrators accountable (many being elderly or deceased), or Somaliland's liminal political position, justice was rarely discussed as an attainable or an exclusively legal concern. Instead, families highlighted the need to teach the younger generations about the atrocities, inform the historical record, and teach the international community about the Somaliland atrocities. According to one man, informing world history was the most important facet of the forensic investigation, a more chief concern than any other transitional justice measure:

‘If they can’t find the perpetrators and put them up for trial … the world finding out is more than enough sentence. The younger generations finding out is more than enough sentence, it is history being recorded and put into a file and recognized for what had happened, recognized for what it was. Even if there is not any more help for those who have died, it is help for the ones who are living and their families. We are more than happy to see it being recorded, being figured out. Being put into record for the world to see, to know that what happened was an atrocity.‘^13^

Nearly all family members mentioned the importance of remembering and communicating to the world what happened during the conflict, emphasizing how little the world knows about Somaliland.

### 5.10 Truth-telling

Truth-telling, as associated with the dead and their deaths, was discussed with 48 individuals. There was too much overlap between answers for a clear breakdown of responses, though there were some general trends in views among the family members. The majority believed that it would be useful to establish a truth-telling forum to share what happened to victims during the war. About half believed that this would be a traumatizing experience, though still a needed exercise. Some, however, did not see any reason for such practices and rather found it superfluous or even detrimental. For example, one market saleswoman believed that truth-telling would be traumatizing, and since everyone already knew what had happened, there was no point in further discussion. Her husband had been captured and shot by the soldiers in 1988 and his remains had been exhumed and reburied under the WCIC. While she did not find discussions important, she did believe that the forensic investigation was important for informing the history of the country and teaching the younger generations about the war. Similarly, several individuals believed that truth-telling was important primarily to inform the country's historical narrative (one individual specifically mentioned school history curricula), to teach younger generations who were not alive at the time, and to inform the rest of the world about the Siad Barre regime's atrocities.

Two individuals were supportive of truth-telling forums but also emphasized the need for legal processes, indicating that truth-telling would not be enough unless the perpetrators were also held accountable. An additional three family members believed that what happened during the wars should not be discussed openly, or was better left for Allah to judge. The tensions between Somaliland and Somalia were also brought up in response to the term ‘truth and reconciliation commission’. Some family members would emphasize that truth-telling would be beneficial, but they were not interested in a Truth and Reconciliation Commission like that in South Africa, since they were not seeking reconciliation with Somalia. For this reason, one practitioner, a Sheikh and local community involved SNM veteran, believed that a truth panel or an institution mimicking the South African would be useful in Somaliland, but that perhaps it should be labeled a truth and forgiveness commission.

Although many believed it was important to remember and discuss what happened during the war, they also believed that it would be traumatizing. Survivors would often discuss their own mental health needs during interviews, or those of their kin, and how there were few resources in Somaliland to assist with lasting traumas from the war. To some, however, engaging in more truth-telling was taboo or culturally inappropriate, partly due to the pact of silence that was agreed upon during the 1990s Somaliland reconciliation conferences [4,47,54]. There were also fears among Isaaq and non-Isaaq families that truth-telling would lead to adverse consequences – like violence against Somaliland minority clans and renewed conflict. Nonetheless, the vast majority were adamant that formal legal processes were needed to address injustices arising from the war; that truth-telling forums would be helpful for communities in their healing processes; and, foremost, that a central part of forensic investigation was to inform the historical record.

## 6 Discussion & conclusions

### 6.1 Survivors’ agenda for transition

Amongst family members of those who were killed under the Siad Barre regime interviewed as part of this study, a majority wanted to find their dead loved one and hold perpetrators accountable, despite many believing this was not a possibility due to the decades that had passed since the conflict. Family members also wanted the stories surrounding the dead to be documented, remembered, and shared nationally and internationally. Additionally, family members focused on formal legal processes primarily emphasized punitive measures against the responsible army commanders (not the foot soldiers) as determined by international tribunals, though in accordance with Shari'a principles. The importance of Shari'a-based law for a transitional justice framework has also been documented in Southern Somalia. A recent study found that several Somali communities outside of Somaliland also believe that Shari'a-based criminal laws need to be incorporated when targeting grievances from the Siad Barre regime and the ensuing decades of civil unrest. Using a survivor-centered approach, the study conducted by Hoehne and Abdullahi [55] indicates that Somalis find Shari'a-based law ideal to establish individual accountability, while the Islamic criminal law courts are seen as less corrupt than other legal institutions. As such, survivor-centered legal processes in Somaliland should ideally incorporate Shari'a-based principles of criminal law. However, as Hoehne and Abdullahi point out, no transitional justice framework to date has included Shari'a alongside other bodies of law; thus, the Somali context would require novel discussions and accommodations [55].

As related to the general need for remembrance, family members were supportive of truth-telling forums, although hesitant that such practices would be retraumatizing for survivors. In addition, while families believed there were many economic needs in Somaliland, including a lack of physical and mental healthcare resources, few emphasized the need for monetary reparations for survivors. Some even believed it could cause more conflict if monetary compensation was granted to some over others, depending on the type of scrutiny compensation would be based on. Rather, family members believed there needed to be more investment in Somaliland institutions to assist all who continued to be burdened by the emotional and economic impacts of the war. Many also found that the recovery of human remains was tied to Somaliland's right to international recognition. Generally, the survivors' agenda for transition focused on four central needs: justice, documentation and remembrance, identification, and social support, none of which have been adequately addressed by the human remains recovery operations. However, there was a recurring hesitancy amongst the non-Isaaq, but also the Isaaq, toward the investigations on the grounds that it can spark renewed conflicts between clans, including more violence and more deaths.

### 6.2 Implications for policy

In Somaliland, forensic investigations are pursued primarily as humanitarian interventions to restore dignity and properly rebury the dead, although the current state of post-conflict affairs precludes formal legal processes and individual identification measures, and there has been limited wider community engagement [4,20]. However, families interviewed as part of this study were looking to find their loved ones and had clear goals associated with the human remains recovery operations, including formal legal processes to hold perpetrators accountable and documentation for the purpose of remembrance. The majority of family members interviewed as part of this study also indicated that they would support truth-telling processes that could help inform the historical record, while urging for further investments in physical and mental health resources for all who become retraumatized when the atrocities are brought up and discussed in their communities, such as when mass grave exhumations are conducted. This implies that further post-conflict processes and transitional justice measures should be pursued alongside the human remains recovery operations as the humanitarian model is not responding to family needs (raising the question of whether they are even ‘humanitarian’). Transitional justice mechanisms discussed by families included further international legal processes to hold army commanders accountable (preferably following Shari'a principles) and (or alternatively) truth-telling forums and informing the historical record, more public documentation of atrocities, investments in social support for survivors, and recovery operations that include individual identification.

Moreover, this study shows that there are several anxieties related to the potential for renewed conflict between the clans, should there be more large-scale mass grave exhumations or further related transitional justice efforts, including truth-telling or reparations. Non-Isaaq clan members have different priorities than many Isaaq family clan members, tied to both fears of aggression from the more populous Isaaq clan and lack of faith in fair trials for crimes committed by the SNM fighters. This implies that further transitional justice processes targeting the Siad Barre era violence need to balance several different interrelated goals, while taking into consideration risks of renewed conflict, retraumatization, and the safety of non-Isaaq as well as Isaaq clan members.

### 6.3 Conclusion

The impact of forensic investigation during and after conflict has been debated since its early use in Argentina in the 1980s, through the subsequent international criminal tribunals of the 1990s and 2000s, and beyond. Whether pursued for humanitarian reasons, for fact-finding, or evidence collection, forensic work, especially that which involves exhumations of mass graves, has had a tendency to impact communities in unforeseeable ways and reach beyond the intended audience or initial goal. That which is legal impacts families and communities even in the absence of their involvement [1], that which is humanitarian can still impact political and legal processes [6], and there are seemingly endless ways in which human remains, justice, memory, and mourning coalesce, inform, and shape one another [22,34,35,63]. As defined by Rubin (pp.119-120) in his analysis of forensic investigations into Francoist violence in Spain:

“(…) incorporation of local perspectives is not simply a matter of reconciling complementary or even competing value systems. If victims' needs and desires are to be taken seriously, those who implement and comment upon transitional justice mechanisms will have to be open to the creative reinterpretations of the techniques and principles that define the field” [12].

The majority of the 74 family members of the dead, interviewed as part of this study, wanted to find their lost loved ones, but they also wanted to hold perpetrators accountable and to preserve the memory of the dead and document the violence. While many would accept documentation and informing the young, the history books, and the international community about the atrocities, there are also deep-rooted calls for a formal legal process with a clear agenda: holding army commanders accountable in international forums in accordance with Shari'a-based criminal law principles. The human remains were also tied to personal traumas and risks of retraumatization, while exhumations were believed to potentially fuel new conflicts between Somaliland clans. Some even believe that no forensic effort or even discussions of the violence should be pursued in Somaliland, as it would inevitably lead to more violence.

This study shows that human remains recovery operations in Somaliland should include individual identification efforts and additional legal and documentation processes to meet survivors' needs. Families have clear agendas for who should be held accountable, how they should be held accountable, and which aspects of society require further investment to address the lasting effects of the atrocities. While these results may have overlap with communities in similar contexts, there is no one-size-fits-all approach to forensic investigation between contexts or even within contexts – family needs are informed by personal experiences as much as cultural and psychosocial needs, and they transform with changing circumstances, understandings, and shifting priorities. Hence, the work presented here can be seen as a starting point for discussing and deliberating upon family-centric forensic needs in Somaliland rather than a final evaluation.

In a context like Somaliland, where there is little funding for forensic investigations or other post-conflict and transitional justice efforts, a mass grave exhumation is a costly undertaking. There needs to be outside knowledge, funding, and expertise, as well as equipment and logistical support. It could be argued that in this context, where there are barely enough resources to open a grave using forensic means, the survivors should perhaps not be led to believe that there could be institutional support for mental health resources, legal processes, or more advanced identification efforts. Moreover, most people in Somaliland lost someone during the conflict, as the author was told on multiple occasions, and many don't know where they were buried. In such a context, investigating who committed a crime and holding them accountable (over 30 years later), let alone finding and identifying the dead, may seem near impossible. Nonetheless, if there is to be a mass grave exhumation, the families of the dead need to be involved in the work or, at a minimum, communities sensitized while managing expectations. These are not efforts that require substantial funding, although some institutional backing and planning for execution may be necessary. Moreover, by engaging with the families in Somaliland, it became apparent that there are tensions surrounding the exhumations, tensions that could contribute to renewed cycles of violence. It was even suggested by some that there had not been community sensitization due to the volatile nature of the work. However, without sensitization, rumors are free to spread, which they have in Somaliland,^14^ and families do not get to weigh in on the work, whether to protest or to share their needs, which may or may not be supported depending on the trajectory of the work and conduct of the forensic practitioners and associated state representatives.

Families in Somaliland are interested in formal legal processes–and with clear agendas for their incorporation--although with little faith in such efforts, they highlight documentation, announcing culpability, truth-telling, and discussions, and mitigating lasting physical and psychosocial effects from the war. Moreover, needs are underpinned by political views (not least the pursuit of Somaliland's international recognition) as well as fears of retraumatization and renewed conflict. While the specific needs identified here are unique to the context and experiences of families of the dead in Somaliland, they may inform needs in other contexts or provide a contrasting basis for evaluating differences across cases. Either way, the results presented here should be incorporated into continued evaluations of family needs and post-conflict processes in Somaliland, and they shine light on the continued need for survivor-driven forensic processes and holistic, integrated approaches to transitional justice.

In 2002, Stover and Shigekane wrote:

“we need to develop a new, more comprehensive strategy for identifying and treating the remains of the dead in the aftermath of war, a coordinated strategy that satisfies both the humanitarian needs of the families of the missing and the legal needs of international war crimes tribunals” [1].

This work has been added to over the past two decades plus, with an emphasis on survivor-, victim-, or actor-centered approaches, such as the work by Simon Robins in Nepal [2], integrated forensic approaches to post-conflict investigations in Uganda [22,34], and grassroots evaluations of forensic processes in Zimbabwe [43,58,64]. This article seeks to add to this body of knowledge with another snapshot in time of family-centered needs. This work aligns with that of many other scholars, showing the context- and culturally specific, experience-based needs of families related to forensic investigation after conflict. This work is not theoretically new nor a counterpoint to the argued de-linkage of retributive and restorative justice through forensic investigation. It is an addition to an existing body of knowledge emphasizing a complex interrelatedness as much as it is a local starting point for more multifaceted work to be continued in Somaliland and elsewhere.

## Ethical review board approval number

UTK IRB-18-04876-XP.

## Declaration of competing interest

The authors declare that they have no known competing financial interests or personal relationships that could have appeared to influence the work reported in this paper.

## Footnotes

1 Identification efforts have only been attempted by use of identifying macroscopic features, e.g., gold teeth, skeletal anomalies, or healed traumas. There has not been any use of molecular methods such as DNA to individually identify the dead.

2 For more information on these components see Ref. [20] Elgerud LMMK. WE HAVE REMAINED PATIENT: FORENSIC HUMAN RIGHTS ANTHROPOLOGY IN POST-SIAD BARRE SOMALILAND: University of Tennessee Knoxville, USA; 2022.

3 The Center for Justice & Accountability (CJA), ‘CJA, Where We Work: Somali/Somaliland’, CJA, http://cja.org/where-we-work/somalia/(accessed 14 February 2023).

4 Elgerud participated in this work in 2014 as a volunteer and helped lead the laboratory analysis of the exhumed human remains.

5 Personal interview, 30-year-old man, Hargeisa, Somaliland, 4 September 2019.

6 Ibid.

7 Personal interview, 60-year-old woman, Hargeisa, Somaliland, 9 October 2019.

8 Personal interview, 61-year-old man, Hargeisa, Somaliland, 19 November 2019.

9 Personal interview, 36-year-old woman, Boroma, Somaliland, 11 December 2019.

10 Personal interview, 30-year-old woman, Hargeisa, Somaliland, 29 September 2019.

11 Personal interview, 34-year-old man, Hargeisa, Somaliland, 29 October 2019.

12 Personal interview, 42-year-old man, Boroma, Somaliland, 11 December 2019.

13 Personal interview, 51-year-old man, Hargeisa, Somaliland, 22 August 2019.

14 For more information see Ref. [20] Elgerud LMMK. WE HAVE REMAINED PATIENT: FORENSIC HUMAN RIGHTS ANTHROPOLOGY IN POST-SIAD BARRE SOMALILAND: University of Tennessee Knoxville, USA; 2022.
